# Climate change could reduce and spatially reconfigure cocoa cultivation in the Brazilian Amazon by 2050

**DOI:** 10.1371/journal.pone.0262729

**Published:** 2022-01-18

**Authors:** Tassio Koiti Igawa, Peter Mann de Toledo, Luciano J. S. Anjos

**Affiliations:** 1 Graduate Program in Environmental Science—PPGCA, Institute of Geosciences, Federal University of Pará—UFPA, Belém, Pará, Brazil; 2 Earth System Science Center—CCST, National Institute for Space Research—INPE, São José dos Campos, São Paulo, Brazil; 3 Parauapebas Campus, Federal Rural University of the Amazon—UFRA, Parauapebas, Pará, Brazil; Universidade de Vigo, SPAIN

## Abstract

Cocoa is a plant with origins in northwestern South America with high relevance in the global economy. Evidence indicates that cocoa is sensitive to a dry climate, under which crop production is reduced. Projections for future climate change scenarios suggest a warmer and drier climate in the Amazon basin. In this paper, we quantify the potential effects in cocoa production due to its edaphoclimatic suitability changes to the Brazilian Amazon biome and account for regional differences in planning occupation territories. We modeled the suitability of cocoa’s geographical distribution using an ensemble of 10 correlative models that were run in the “biomod2” library and projected to two future climate scenarios (RCPs 4.5 and 8.5) by 2050. Combining information on climate and soil suitability and installed infrastructure in the macro-regions of the Brazilian Amazon. We defined a zoning system to indicate how cocoa production may respond to climate change according to the current and future suitability model. Our results suggest that a reduction in precipitation and an increase in temperature may promote a reduction in the suitability of cocoa production in the Brazilian Amazon biome. In addition of the areas suitable for cocoa plantation, we found a 37.05% and 73.15% decrease in the areas suitable for intensification and expansion zones under RCP 4.5 and 8.5, respectively, compared with the current scenario. We conclude that there may be a need to expand land to cocoa production in the future, or else it will be necessary to plant a cocoa variety resistant to new climatic conditions. Besides, we recommend procedures to combat illegal deforestation to prevent the most critical climate change scenarios from occurring.

## Introduction

Agriculture is one of the human activities that is are most vulnerable to climate change [1]. Some studies have indicated that such a threat can reduce global agricultural production [2, 3]. According to [4], the reduction in crop yields is predicted to be between 10% and 25% in 2050. Consequently, the reduction of food supply will increase food prices and food insecurity, especially in the world’s poorest regions [5].

In this context, we can include cocoa (*Theobroma cacao* L.), which is considered an essential crop in several tropical countries because of the supported income it provides many small farmers [6]; it is also involved in a global chain of chocolate production. South America represents 12.49% of global cocoa production and its countries receive US$2.4 billion/year [7] through its exportation. Brazil is the largest producer of cacao in South America and the sixth-largest producer globally, with a production of 235 809 tons/year [7]. The Brazilian Amazon biome represents 52.12% of cocoa production in Brazil [8]. According to the last agricultural census [9], climate change may directly affect the income of more than 20 550 families. Therefore, it is possible to observe the importance of carrying out studies in order to identify how climate change will change the future spatial configuration of cocoa production in the region.

From a biogeographic point of view, cocoa is a plant native to northwestern South America, where it originally evolved. It has become thoroughly adapted to a long history of domestication by humans initiated 5300 years ago [10]. Cocoa preferably grows in regions with rainfall ranging between 1400 and 2000 mm/year [11] and temperatures above 21°C. In general, its development occurs in hot and humid climates with a latitudinal range of 20°N to 20°S [12]. According to some studies, climate change can affect the cocoa growing zones [6]. In drier conditions, cocoa growth without intercropping may have a lower mortality rate than when planted in conjunction with another plant because it avoids competition for water with other crops [13].

However, the agricultural sector is one that is most vulnerable to the impacts of climate change [14]. Thus, in the current scenario of potential consolidation of a non-analogous climate, studies that estimate the possible impacts on agricultural production chains caused by climate change are of great importance. Some projections regarding South American biomes also indicated the persistence of an annual temperature rise over a long period and a significant reduction in humidity in forest biomes [15]. In this sense, Brazil has carried out several projects to promote low carbon rural development by maximizing conservation and economic production in the region, such as the Legal Amazon Ecological–Economic Macro-Zoning (MacroZEE) [16].

The MacroZEE consists of a project carried out in the Legal Amazon to encourage the reduction in regional inequality, stimulate sustainable development in the region, and delimit the regions in the Amazon biome based on socio-environmental and economic characteristics. The territories were classified into three main categories: the network territory, zone territory, and border territory [17]. The network territory consists of areas within consolidated occupations with extreme mobility, where flows and connections are susceptible to overlap. The predominance of preserved ecosystems characterizes the zone territory. The border territory consists of areas with different levels of land appropriation, settlement, and consolidation [17].

However, despite the existence of projects with the purpose of contributing to sustainable development in the Brazilian Amazon biome. There is a lack of studies regarding the impacts that climate change may have on the agricultural sector in the region. Therefore, the objective of this study was to identify and quantify the zones of adaptation to climate change to support territorial planning using species distribution modeling methodologies.

## Materials and methods

### Study site

The Brazilian Amazon biome is one of the most significant areas of cocoa production in Brazil. Also, the genus displays the highest taxonomic and genetic diversity in this biome. Therefore, the Amazon biome is considered to be the center of origin of *T*. *cacao* L. [10]. The study area is one of the most significant areas of cocoa production areas in Brazil, representing approximately 50% of national production [8]. The region comprises an area of 4 186 943 km^2^, which represents 49% of the national territory [18]. Rainfall in the Amazon has an annual seasonality that varies between 2.2 to 2.6 mm/day in the driest months (July and August) and reaches 9 mm/day in the rainiest months (February and March) [19].

### Datasets

Species occurrence data were obtained from “SpeciesLink” [20] and the Global Biodiversity Information Facility [21]. The raw data underwent a process of cleaning (1310 occurrence points) during which the miswritten (wrong values for geographic coordinates) and duplicated coordinates were removed. Consequently, the number of occurrence points was reduced from 1310 to 288 (S1 Table).

The occurrence points of cocoa are also distributed in regions outside the Amazon biome (S1 Fig), such as the state of Bahia which, according to Mendes (2018), in 1746, cocoa seedlings were brought to this state by farmer Louis Frederic Warneaux. Currently, the state of Bahia is the second largest national producer with 113,039 tons, which represents 43.57% of Brazilian production [8]. Therefore, the occurrence points that are located in the state of Bahia are of rural properties. We are aware that occurrences in plantations may lie outside the species climatic niches. Nevertheless, as our goal is related to cocoa cultivation we opted to use those occurrences in the modelling process.

### Climate variables selection

The 19 bioclimatic variables were obtained from climatologies at high resolution for the earth’s land surface areas [22]. Then, we clipped all data from bioclimatic variables for the South America and we calculated the correlation between all these variables. The collinearity among predictors decreases the efficiency and increases the uncertainty of species distribution models [23]. Therefore, the bioclimatic variables with a Pearson correlation coefficient between two variables greater than 0.7 were eliminated to reduce collinearity. Thus, from the selected variable, only six climatic variables obtained a correlation coefficient below 0.7. The six selected variables were: mean daytime variation (°C), isothermality, average temperature of the hottest quarter (°C/10), average temperature of the driest quarter (°C/10), precipitation of the driest month (mm/month), and the seasonality of precipitation (coefficient of variation).

To generate the future species distribution model, we used representative concentration pathways (RCPs) 4.5 and 8.5 from the six selected bioclimatic variables. These RCPs simulate changes based on the set of anthropogenic forcing scenarios related to the concentration of greenhouse gases in the future [24]. The five GCMs (2041–2060) chosen for RCP 4.5 and 8.5 scenarios were as follows: CESM1-BGC, generated by the National Center for Atmospheric Research [25]; CMCC-CMS, created by Centro Euro-Mediterraneo per i Cambiamenti Climatici [26]; CM5A-LR, prepared by the Institut Pierre-Simon Laplace [27]; MIROC5, generated by the University of Tokyo [28]; and ESM-MR, created at the Max Planck Institute for Meteorology [29]. According to [30], we selected the models that allowed to achieve the maximum independent ensemble quality.

The future cocoa distribution models were created by making a consensus model that consist of the average of each bioclimatic variables. This procedure reduces the uncertainties created by using only one general circulation model [31].

### Species distribution model

We used the biomod2 library [32] from R [33] to create the species distribution model. Furthermore, we used 300 pseudo-absence points that we created using the Surface Range Envelope strategy. As a result, it was possible to generate pseudo-absence points in places outside the climatic variation of the occurrence points to improve the accuracy of the samples [34].

For each scenario (RCP 4.5 and 8.5), the Cocoa distribution was analyzed using the ensemble method from ten species distribution modeling algorithms: Classification and Regression Trees [35], Generalized Boosted Regression Models [36], Random Forest [37], Generalized Linear Models [38], Generalized Additive Models [39], Multivariate Adaptative Regression Splines [40], Flexible Discriminant Analysis [41], Surface Range Envelope, MaxENT [42], and Artificial Neural Network [43].

The models were validated using 25% of the samples and the others 75% were used to training the models. We generated an ensemble of the different algorithms by calculating the arithmetic average of those algorithms with a TSS higher than 0.7 [44].

### Soil suitability classification

Soil type is an important variable that identifies a suitable location for planting certain crops. From this point of view, previously reported studies have identified that the Amazon basin is mainly characterized by soil of low fertility [45]. The addition of mineral fertilizers is necessary to meet the demand for cocoa [46]. However, there are few mines or factories in close proximity to the Amazon basin, which means that transporting fertilizers to the region is costly [47].

We identified low stony soil (soils with a small amount of gravel that are coarse elements, with a length above 1mm) as suitable for cocoa plantation. Suitable soils were further classified based on their fertility. Soils with high fertility were characterized as eutrophic, which means that at least 50% of their cation exchange capacity is occupied by nutrient cations [46]. Therefore, soils with low fertility were characterized as dystrophic, which means that less than 50% of the cation exchange capacity is occupied by nutrient cations.

Soils with high fertility can be used for planting cocoa in areas with less infrastructure support as they require lesser amounts of chemical fertilizers, which makes production costs cheaper. However, in more isolated regions (the zone territory), it is necessary to conduct a more detailed analysis of the economic viability of the plantation, mainly due to the costs associated with production flow. Finally, soils with low fertility must be located in regions with good infrastructure support, which decreases the cost of transportation. We used the network territory created in the MacroZEE project to delimit the region with extreme mobility.

The economic viability of these less fertile soils was based on current cocoa production in the Amazon region. According to the cocoa production data [8] associated with these soils with low fertility, municipalities that do not have fertile soils represented 21.94% of the entire production in the region. This shows that it is possible to plant cocoa in places with less fertile soils. Therefore, soils characterized as suitable for cocoa planting were those with high fertility in all territories of MacroZEE or soils with low fertility that were located within the network territory. This procedure was carried out to promote a delimitation of soils based on the relationship between infrastructure and soil fertility, as they are linked to the economic viability of the plantation.

We used the soil data obtained from the Brazilian Institute of Geography and Statistics website [48]. We chose the IBGE database because it has a finer classification resolution compared to other global databases. Thus, the soil types were selected according to the world reference base, i.e., the eutrophic and dystrophic subtypes of Acrisols, Fluvisols, Nitosols, and Ferralsols.

### Data analyses

For the analysis of the suitability data, we clipped the consensus model for the areas with soil suitable for cocoa plantation in the Amazon biome region in order to quantify the edaphoclimatic adequacy at a more detailed mapping level. We associated these results with the territories of MacroZEE. Then, we calculated the analysis of variance of MacroZEE territories and RCP scenarios. It is worth mentioning that the selected areas were outside indigenous lands and conservation units.

To carry out the creation of mitigation measures to reduce the problems caused by climate change, it was necessary to perform a detailed analysis based on the categorization of current and future edaphoclimatic suitability of cocoa, and the identification of cocoa producing areas Therefore, we adapted the classification method for these zones elaborated in the work of [49] for the zoning of suitable areas for cocoa plantation in the Brazilian Amazon biome, as shown in Table 1. The zoning for the current and project climatic conditions was created based on the criteria of the current prevalence of cocoa cultivation column and climates suitability columns (Table 1).

**Table 1:** Schematic zoning of the Brazilian Amazon biome.

Zone	Current climatic suitability	Projected future climatic suitability	Current prevalence of cocoa farming	Focus of adaptation strategy	Key requirements	Local or regional examples
Intensification zone	Medium to high (>50%)	Medium to high (>50%)	(Co-)dominant crop in local farming systems	Improvement of production techniques and inputs to increase productivity; diversification of production to protect against environmental and market risks	Technical assistance; input supplies; affordable credit	Municipalities of Uruará, Medicilância and Altamira located in the state of Pará
Expansion zone	Medium to high (>50%)	Medium to high (>50%)	Present, but not dominant in local farming systems or Absent.	Expansion in deforested areas combined with forest conservation	Monitoring and inspection of government agencies ensuring land use planning and resource conservation; technical assistance; affordable credit	Most of the state of Rondônia
Diversification zone	Low to medium (20 to 50%) or Medium to high (>50%)	Low to medium (20 to 50%)	(Co-)dominant crop or present, but not dominant in local farming systems	Diversification of rural properties with crops or varieties more resistant to heat and drought; use of techniques to promote lesser impacts to crops caused by changes at the microclimate level.	Consolidated suppliers with a wide variety of products; Government incentives for tree production; technical assistance; affordable credit	Municipalities of Cumaru do Norte located in the state of Pará
Conversion zone	Medium to high (>50%) or Low to medium (20 to 50%)	Very low (<20%)	(Co-)dominant crop or present, but not dominant in local farming systems	Progressive transition through diversification with resistant crops and supply chains better adapted to future weather conditions	Suppliers with products adapted to alternative cultures; technical assistance; affordable credit	Municipality of Rio Maria located in the state of Pará
Not Recommended Zone	Medium to high (>50%) or Low to medium (20 to 50%) or Very low (<20%)	Very low (<20%)	Absent	Not Recommended to cocoa plantation	-	Municipality of Santa Cruz do Xingu located in the state of Mato Grosso

**Fig 1:**
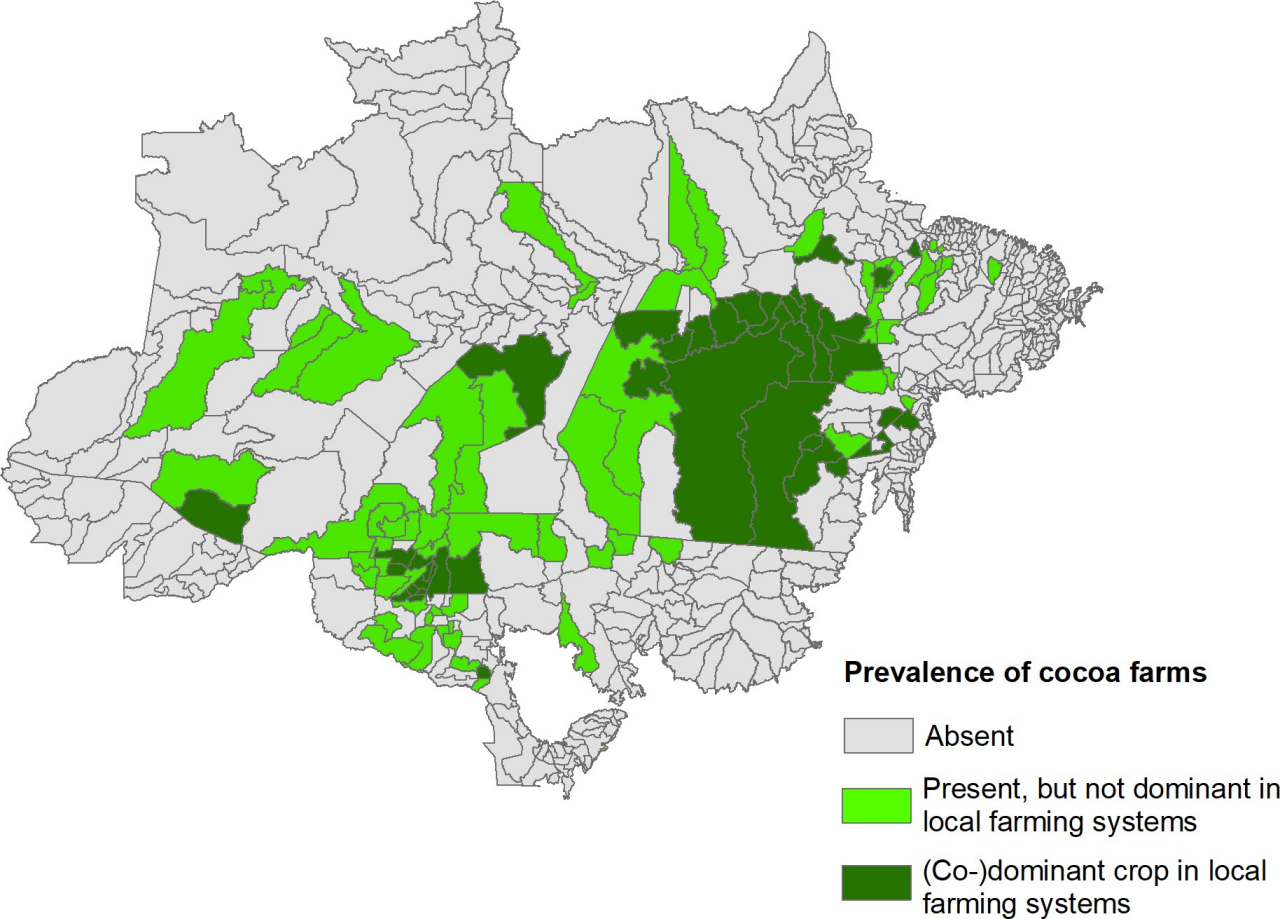
Spatial distribution of the prevalence of cocoa farms.

**Fig 2:**
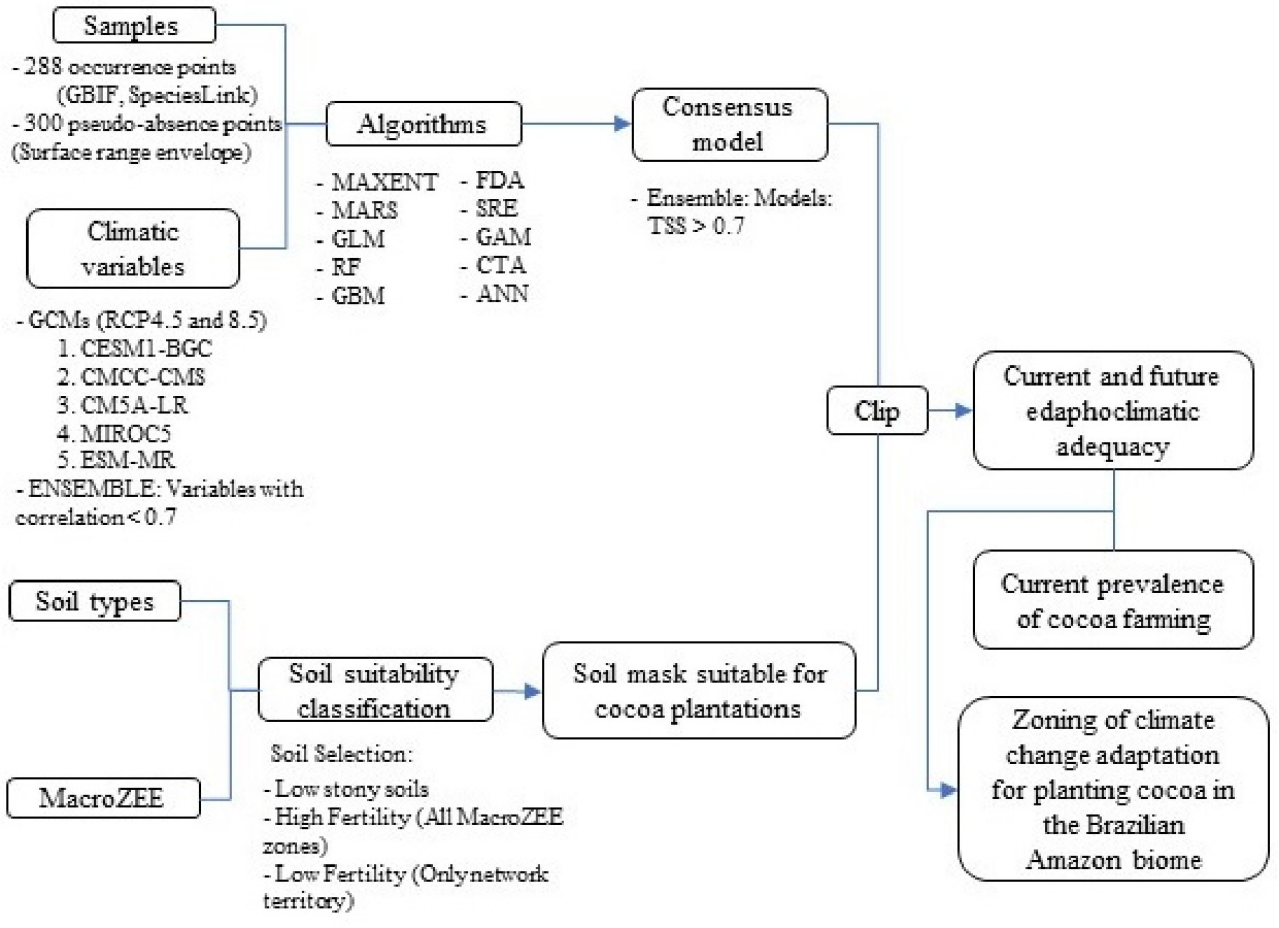
Summary of all methodological procedures.

The current prevalence of cocoa farms was used to elaborate the zoning of the current and future scenarios. The categorization of the current prevalence of cocoa cultivation was based on the analysis of the cocoa production values from the data in [8], in relation to other perennial crops present in the municipalities. Thus, the municipalities characterized as (co-) dominant in local agricultural systems were those where cocoa was first or second in the ranking of production values in relation to other perennial crops. The municipalities considered as present, but not dominant, were those that obtained a position equal to or below the third place. The absent municipalities were those that had no production value. The spatial distribution of the current prevalence of cocoa cultivation can be seen in Fig 1. Finally, the summary of all methodological processes can be seen in Fig 2.

## Results and discussion

The model predictions achieved excellent performance in the validation, as the TSS of the ten models generated was between 0.817 and 0.875. The Area Under the Curve was between 0.908 and 0.986. All models were used to develop the consensus model because they obtained TSS values above 0.7. Concerning the importance of the variables for current cocoa distribution followed the order: mean diurnal range (bio2), mean temperature of the driest quarter (bio9), isothermality (bio3), precipitation of the driest month (bio14), mean temperature of the wettest quarter (bio8), and precipitation seasonality (bio15).The soil suitability classification showed that the soil areas suitable for cocoa plantation represented approximately 17% of the Brazilian Amazon biome (S2 Fig). According to the ensemble of climatic data, it was possible to identify a trend of increasing temperature and decreasing precipitation in areas with soil suitable for planting cocoa. The mean diurnal range increased in the assessed period by 0.25 and 0.31°C in the RCP 4.5 and RCP 8.5 scenarios, respectively. The mean temperature of the driest quarter increased by 2 and 2.67°C in the RCP scenarios 4.5 and 8.5, respectively. The mean temperature of the wettest quarter increased by 1.45 and 2.94°C in the RCP scenarios 4.5 and 8.5, respectively. Precipitation of the driest month decreased by 9 and 19.6 mm/month in the RCP scenarios 4.5 and 8.5, respectively. The precipitation seasonality increased from 4 and 7.7 coefficient of variation in the RCP scenarios 4.5 and 8.5.

The higher temperature depicted in the RCP scenarios hurts cocoa yields [50]. According to [6], high temperature can also cause stress indirectly due to the higher evapotranspirative demand of the air. Thus, some studies recommend the implementation of production technologies to stabilize the temperature of the microclimate and reduce the impacts of high rainfall, such as the use of shading plants and canopy manipulation [51].

The climatic variable of precipitation in the driest month indicated a decrease in precipitation in the future scenarios. Such a dry environment will cause damage to the development of cocoa, considering that it requires a rainfall rate above 1400 mm/year [11]. Specifically, when the deficit of water in the soil increases, the production and growth of the cocoa tree decreases [52]. Furthermore, younger plants are more sensitive to water deficit than older plants. Thus, a prolonged drought period will have a significant impact on cocoa growth [53].

**Fig 3:**
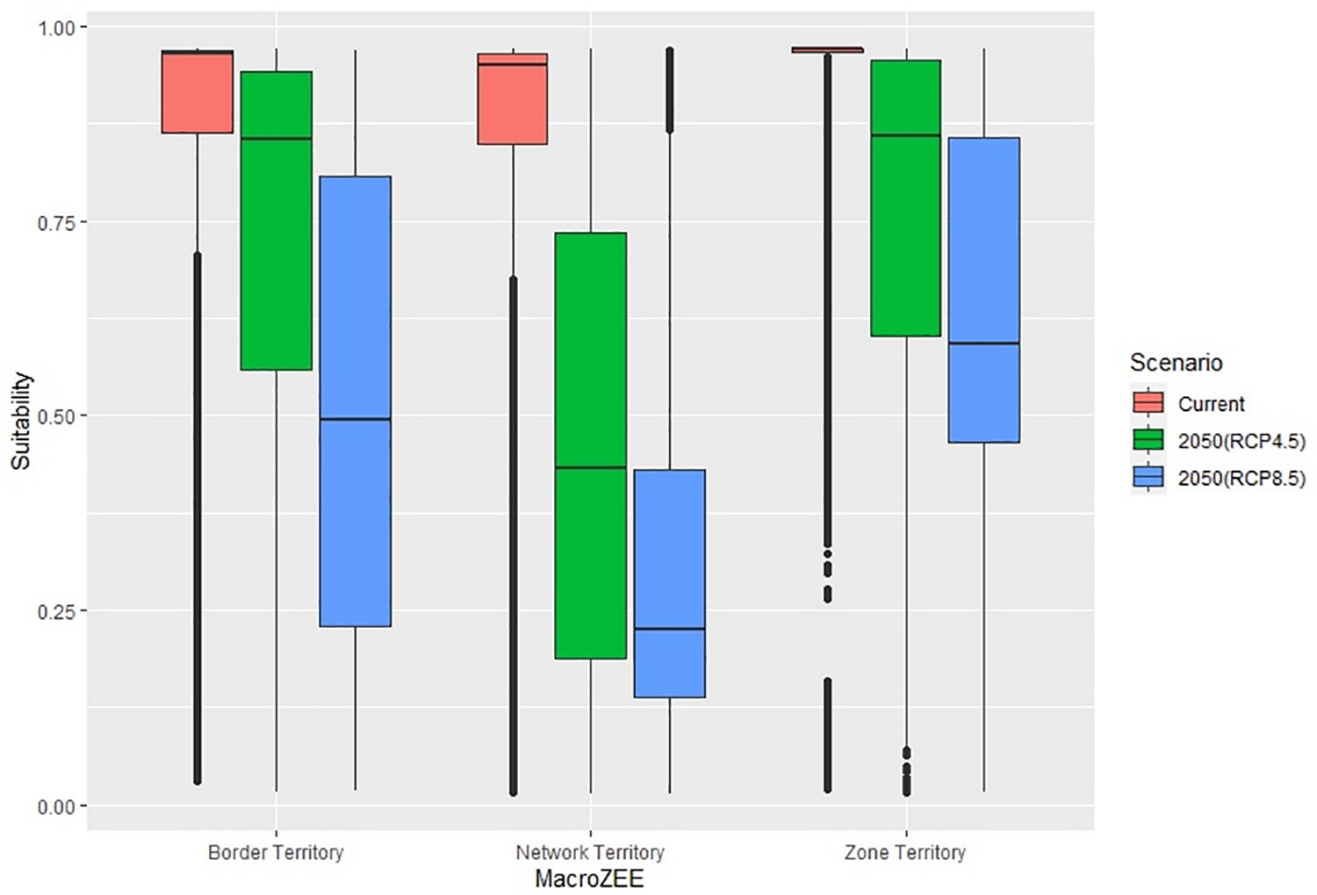
Distribution of current and future cocoa adequacy (RCP 4.5 and 8.5 scenarios) in MacroZEE territories.

According to Fig 3, from the relationship between the edaphoclimatic suitability of cocoa and the territories of MacroZEE, it can be seen that the network territory will suffer the most losses in terms of climatic suitability. From the results of the analysis of variance, it was possible to identify a statistically significant difference between scenarios as well as between territories (both with p<0.001). Furthermore, as shown in Fig 3, the most suitability losses occur under the RCP 8.5 scenario.

The reduction in the suitability of areas for cocoa production identified in this study is similar to that found in another study carried out in Central America [54]. The result was generated by the RCP scenarios that showed an increase in temperature and a reduction in precipitation in the study region. Additionally, the most affected area was within the territory with consolidated economic activities (the network territory).

**Fig 4:**
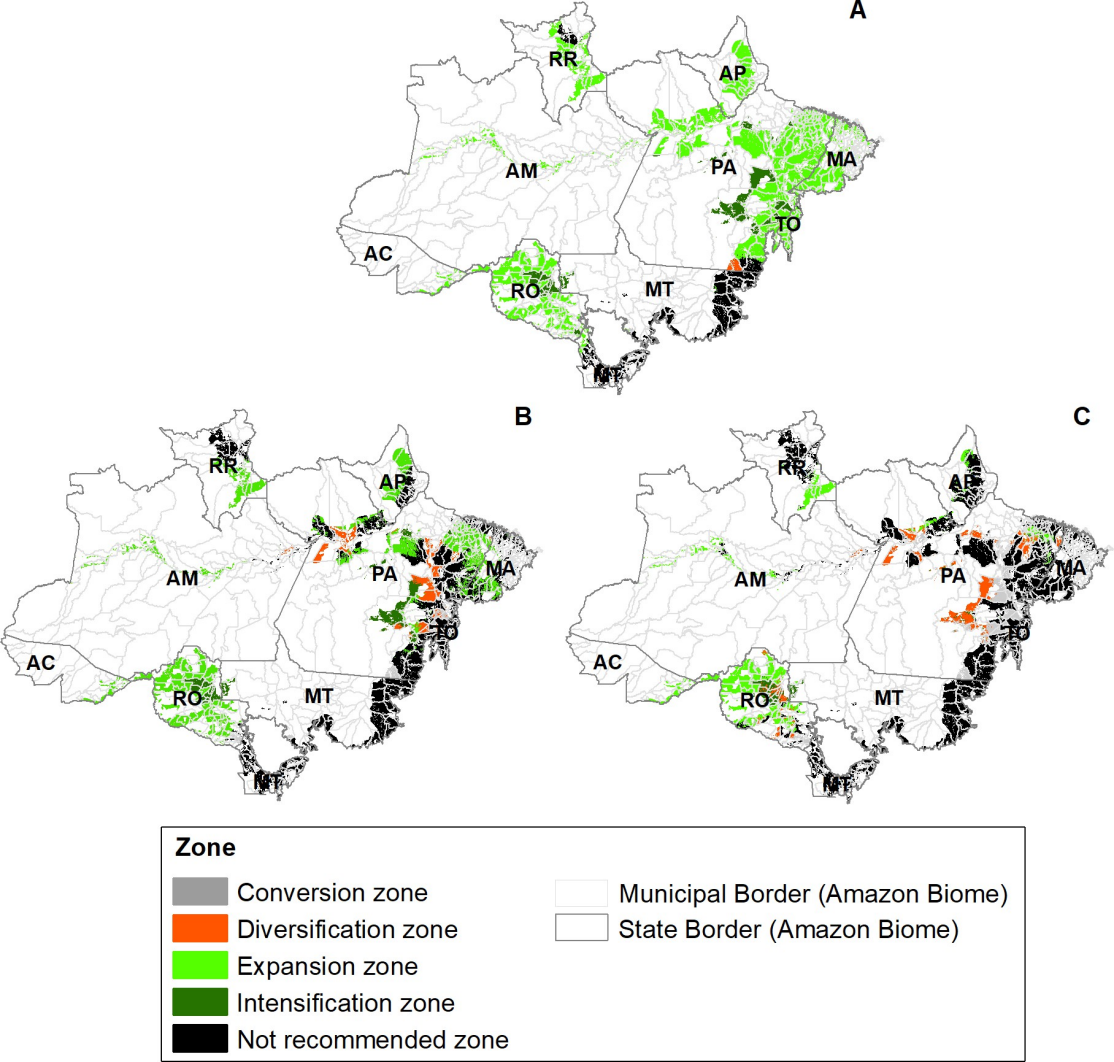
Zoning of climate change adaptation for planting cocoa in the Brazilian Amazon biome (AC–Acre, AP-Amapá, AM–Amazonas, MA—Maranhão, MT—Mato Grosso, PA–Pará, RO–Rondônia,—RR–Roraima and TO–Tocantins). (A) Current, (B) 2050 (RCP4.5), and (C) 2050 (RCP8.5). Map source: Municipal border and state border [48].

Finally, from the spatialization of the climate change adaptation zones for planting cocoa in the Brazilian Amazon biome (Fig 4), it was possible to observe that the largest areas not recommended for cocoa planting are located in the southeast of the state of Pará (PA) in the RCP8.5 scenario and in the RCP4.5 scenario. Likewise, there was an increase in the conversion zones in Southeast Pará in both scenarios of RCPs. Thus, taking into account the current cocoa production. The reduction of conversion zones for cocoa production in Southeast Pará could generate a drop of at most 4.2% in national production [8]. From a socio-economic point of view, it can affect the income of more than 8,823 families. It was possible to infer because, according to the last agricultural census, there are 8,823 cocoa-producing establishments in the southeast of Pará, 77% of which are family farmers, that is, the workforce is made up of the owner’s own family [8]. Furthermore, a large area suitable for expanding cocoa plantation in the state of Rondônia (RO) in both future scenarios was identified. The quantification of these zones for adaptation to climate change is shown in Table 2. It is worth noting that the expansion zones in the RCP8.5 scenario comprise 20,22% less area than that in the RCP4.5 scenario.

**Table 2:** Quantification of the adaptation zones.

Classes	Current		RCP 4.5		RCP 8.5	
	Area (km^2^)	%	Area (km^2^)	%	Area (km^2^)	%
Conversion zone	1597.95	0.21	15505.92	2.08	56593.59	7.61
Diversification zone	4391.77	0.59	46419.80	6.24	64436.06	8.66
Expansion zone	532394.94	71.55	279644.67	37.58	129174.18	17.36
Intensification zone	73834.26	9.92	53157.47	7.14	21657.23	2.91
Not Recommended zone	131843.07	17.72	349334.13	46.95	472200.93	63.46
Total	744061.99	100.00	744061.99	100.00	744061.99	100.00

The quantification of the adaptation zones (Table 2) allowed us to identify that climate change may promote a 36.75% and 61.2% reduction in the areas suitable for intensification or expansion of cocoa plantation under the RCP 4.5 and 8.5 scenarios, respectively, in comparison with the current scenario. Despite this, there will still be a large region available for expansion and intensification in both scenarios, i.e., 44.73% (RCP 4.5) and 20.27% (RCP 8.5) of all areas with soil in the Brazilian Amazon biome suitable for cocoa plantation.

The decrease in intensification zones (Table 2) indicates that the current production areas will suffer from the loss of suitability of cocoa production. Thus, the regions that currently produce cocoa are likely to suffer from the decrease in production, consequently, there will be losses in the production chain. These results corroborate with several other studies that indicated loss of production in many other agricultural crops in Brazil promoted by climate change [55, 56].

In addition, it was observed that a large area needs to implement the transition and diversification process by planting another crop that is more resistant to drought and heat. The diversification and conversion zone has 8.32% (RCP4.5) and 16.27% (RCP8.5) of all areas with soil suitable for cocoa plantation. Furthermore, we observed increases in the not recommended zone of 29.23% and 45.74% under the RCP4.5 and 8.5 scenarios, respectively, compared to the current non-recommended zone.

According to the results, we may observe an increase in diversification and conversion zones. Thus, it is necessary to carry out procedures to reduce water stress because cocoa is considered to be sensitive to water deficit. Although a large part of the cocoa crops are in agroforestry systems, it is still possible to find plantations in full sun in the Brazilian Amazon biome. In this way, one of the main strategies for adapting to climate change in these areas is the adoption of agroforestry systems with the planting of crops that are more resistant to heat and drought (Table 1). According to [50], agroforestry systems can buffer extreme climatic conditions and reduce stress in the cocoa tree. Conversely, some species that are adapted to being the shaded tree species of a cocoa agroforestry system may promote a risk to the functioning of the system under extended severe drought conditions. This can happen because the soil water content in shaded systems is lower than that in systems exposed to complete sunlight, suggesting that cocoa mortality in shaded systems is linked to strong competition for soil water [13]. Therefore, in the case of the implementation of a new species in agroforestry systems, a specific study is necessary to verify whether this species could be harmful when intercropping with the cocoa plantation.

Another method for the mitigation of climate change impacts on cocoa plantations, which avoids the relocation of plantations, is plant breeding. This is a procedure that can select a specimen more adapted to the new climatic conditions. Nevertheless, this adaptation is challenging as it requires a coordinated and long-term approach to maintain this research network in the long run [1] considering that cocoa is a perennial crop and therefore more time is needed to create a new plant variety.

However, reducing carbon emissions from deforestation and forest degradation is an important strategy for mitigating climate change [57]. Therefore, it is necessary to carry out public policies to combat illegal deforestation. Strategies to reduce deforestation include repression through licensing, monitoring and fining procedures. The severity of penalties for deforestation must be sufficient to prevent illegal deforestation, but not so great as to be unenforceable. Policy reform is also needed to address the causes of deforestation, including the role of deforestation in establishing land claims [58]. In this context, the cultivation of cocoa in agroforestry systems is a resource used by farmers in the south of the state of Pará, for the reforestation of deforested areas, and it is an economically attractive option for these farmers [47].

## Conclusions

From the data presented, it is possible to conclude that by 2050, a loss in the suitability of Brazilian Amazon for cocoa planting is likely, owing to a decrease in precipitation and increase in temperature caused by climate change. However, based on the results obtained, there will still be large tracts of land with high levels of edaphoclimatic suitability in the Brazilian Amazon biome.

According to the results of the zoning quantification, it is possible to observe a broad extension of suitable areas for the intensification and expansion of cocoa plantation; expansion zones make up most of these areas. Thus, there may be a need in the future to expand cocoa production, or it will be necessary to develop a cocoa variety resistant to new climatic conditions.

The decrease in intensification zones indicated losses in suitability for the cocoa plantation in the current production areas. Then, this will likely promote a reduction in cocoa production. There are several strategies to mitigate climate change for cocoa plantations, such as the use of agroforestry systems and plant breeding. Besides, the decrease in greenhouse gas emissions contributes to the consolidation of less intense scenarios of climate change. Therefore, policies to combat illegal deforestation through strategies that include repression through licensing, monitoring, and fining procedures are recommended.

## Supporting information

S1 Fig Location map of the occurrence points in the Brazilian Amazon biome. (DOCX)

S2 Fig Soil potentially suitable for planting cocoa. (DOCX)

S3 Fig Current and future climate suitability of cocoa scenarios (a) current, (b) future—RCP 4.5 and (c) future—RCP 8.5.
(DOCX)

S1 Table Occurrence points of the *Theobroma cacao* L. (DOCX)
