# Supplementary figures and images for: Climate change could reduce and spatially reconfigure cocoa cultivation in the Brazilian Amazon by 2050
Source: PLoS One. 2022 Jan 18;17(1):e0262729. doi: 10.1371/journal.pone.0262729 (PMC8765622; doi:10.1371/journal.pone.0262729)

**S1 Figure.** Location map of the occurrence points in the Brazilian Amazon biome


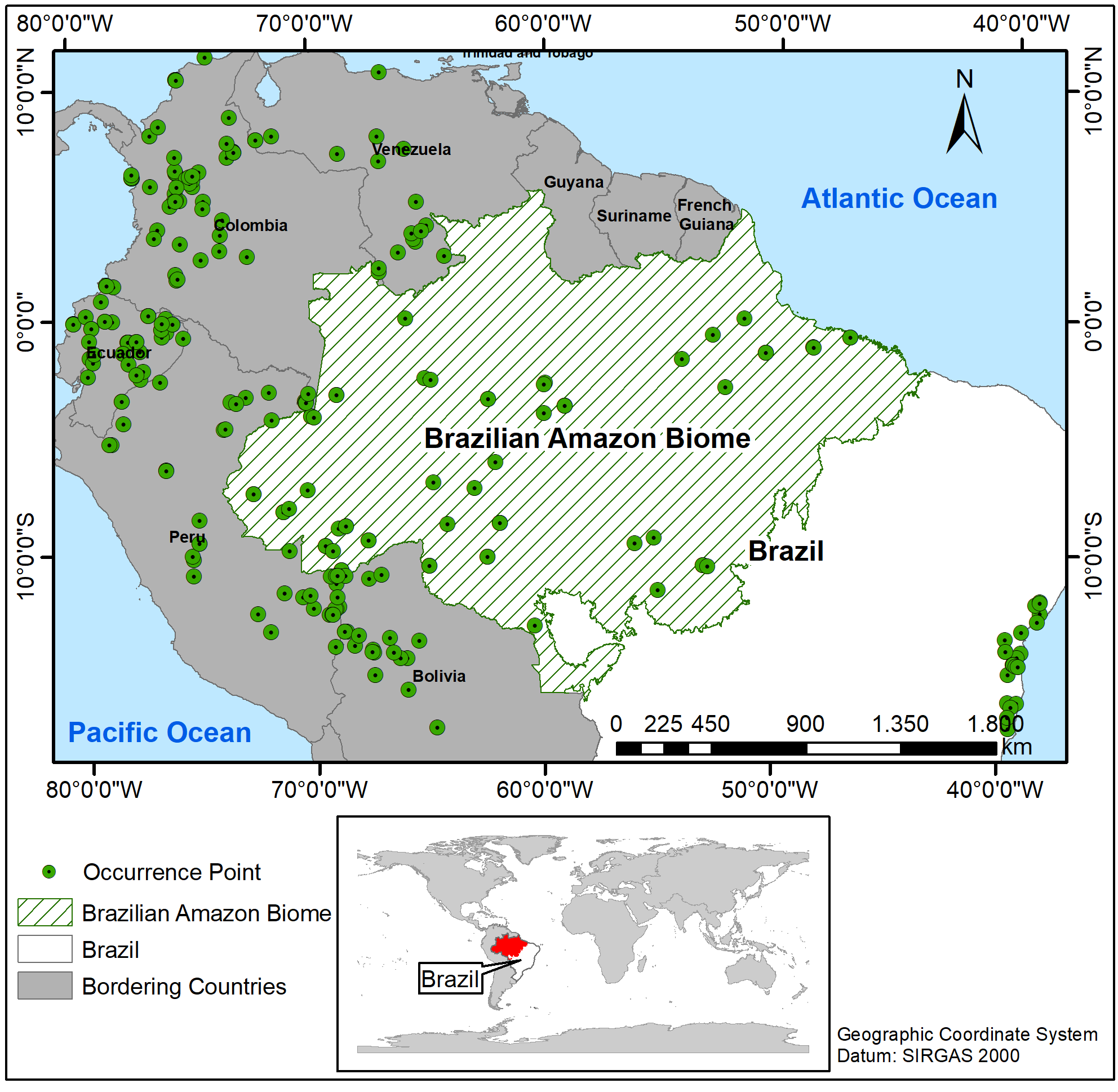

Supplement: S1 Fig — (DOCX) [file pone.0262729.s001.docx]

**S2 Figure.** Soil potentially suitable for planting cocoa


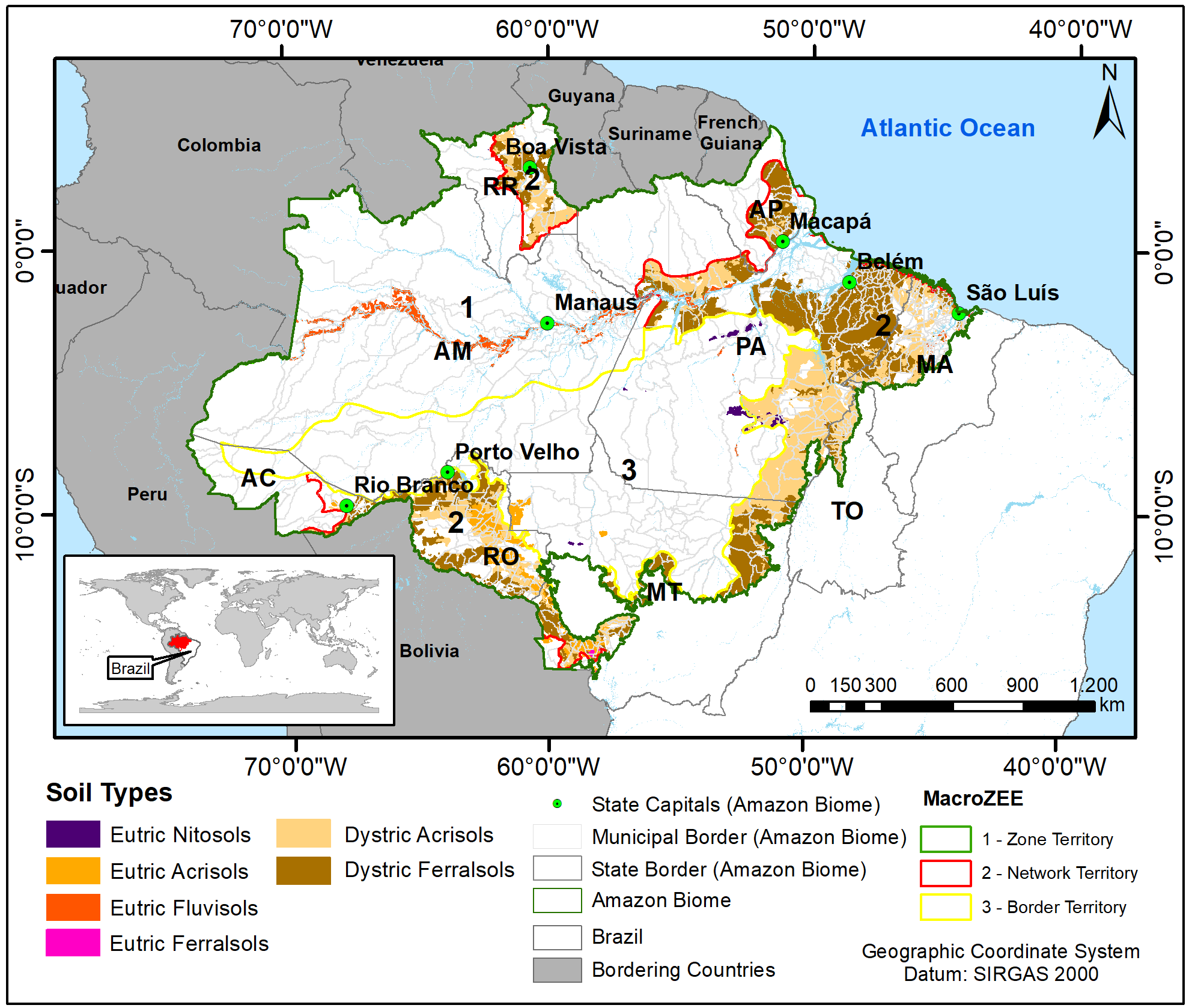

Supplement: S2 Fig — (DOCX) [file pone.0262729.s002.docx]
