# Supplementary material for: Climate change could reduce and spatially reconfigure cocoa cultivation in the Brazilian Amazon by 2050
Source: PLoS One. 2022 Jan 18;17(1):e0262729. doi: 10.1371/journal.pone.0262729 (PMC8765622; doi:10.1371/journal.pone.0262729)

**S3 Figure.** Current and future climate suitability of cocoa scenarios

The result generated from the current and future cocoa distribution model for scenarios RCP 4.5 and 8.5, showed a great reduction in the climatic adequacy of cocoa in the eastern region of the Brazilian Amazon biome (S3 Figure).

**S3 Figure.** Current and future climate adaptation of cocoa scenarios (a) current, (b) future - RCP 4.5 and (c) future - RCP 8.5


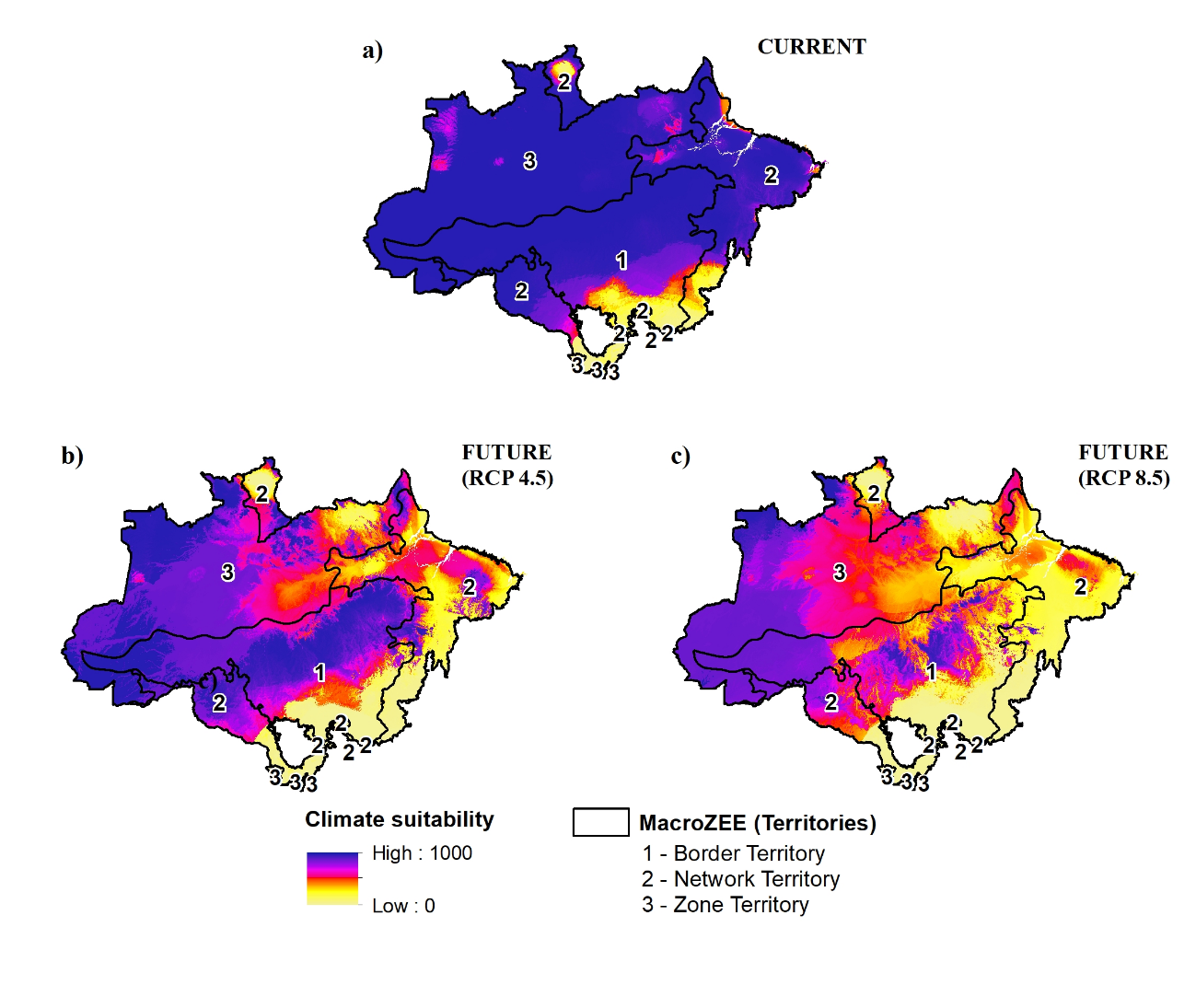

Supplement: S3 Fig — Current and future climate suitability of cocoa scenarios (a) current, (b) future—RCP 4.5 and (c) future—RCP 8.5. (DOCX) [file pone.0262729.s003.docx]
