# Supplementary material for: Climate change could reduce and spatially reconfigure cocoa cultivation in the Brazilian Amazon by 2050
Source: PLoS One. 2022 Jan 18;17(1):e0262729. doi: 10.1371/journal.pone.0262729 (PMC8765622; doi:10.1371/journal.pone.0262729)
Supplement: S1 Table — (DOCX) [file pone.0262729.s004.docx]

**S1 Table.** Occurrence points of the *Theobroma cacao* L.

| Point | Latitude | Longitude |
| --- | --- | --- |
| 1 | 9°40' 48,000" S | 67°39' 0,000" W |
| 2 | 10°56' 30,661" S | 68°51' 8,802" W |
| 3 | 4°22' 5,900" S | 70°2' 16,000" W |
| 4 | 1°25' 12,000" S | 48°27' 0,000" W |
| 5 | 19°56' 10,000" S | 40°37' 0,001" W |
| 6 | 16°22' 40,001" S | 39°34' 48,000" W |
| 7 | 0°57' 0,000" S | 52°45' 0,000" W |
| 8 | 7°36' 59,976" S | 72°36' 0,000" W |
| 9 | 7°37' 0,001" S | 72°36' 0,000" W |
| 10 | 4°30' 0,000" S | 71°45' 0,000" W |
| 11 | 15°10' 0,001" S | 39°35' 0,000" W |
| 12 | 12°30' 0,000" S | 38°18' 0,000" W |
| 13 | 16°23' 15,997" S | 39°11' 1,000" W |
| 14 | 2°48' 0,000" S | 65°8' 0,000" W |
| 15 | 14°47' 0,000" S | 39°10' 48,000" W |
| 16 | 2°0' 29,002" S | 54°4' 9,001" W |
| 17 | 6°24' 22,219" S | 62°6' 5,281" W |
| 18 | 14°10' 1,999" S | 39°43' 19,999" W |
| 19 | 9°2' 13,999" S | 64°12' 2,002" W |
| 20 | 11°52' 23,002" S | 55°2' 53,999" W |
| 21 | 12°9' 37,001" S | 38°30' 20,999" W |
| 22 | 0°57' 50,500" S | 46°52' 42,500" W |
| 23 | 14°13' 13,001" S | 39°3' 34,999" W |
| 24 | 13°19' 13,001" S | 39°3' 37,001" W |
| 25 | 14°25' 53,760" S | 39°13' 26,400" W |
| 26 | 14°43' 0,001" S | 39°22' 57,000" W |
| 27 | 16°33' 37,001" S | 39°25' 13,001" W |
| 28 | 13°40' 49,001" S | 39°46' 50,002" W |
| 29 | 8°59' 59,000" S | 61°55' 12,000" W |
| 30 | 8°59' 58,999" S | 61°55' 12,000" W |
| 31 | 8°59' 59,000" S | 61°55' 12,000" W |
| 32 | 17°30' 4,828" S | 39°30' 43,355" W |
| 33 | 9°52' 32,401" S | 56°3' 55,901" W |
| 34 | 0°14' 24,299" S | 51°24' 45,900" W |
| 35 | 3°11' 55,021" S | 52°12' 44,420" W |
| 36 | 10°48' 20,120" S | 65°0' 28,631" W |
| 37 | 1°40' 55,992" S | 50°28' 49,080" W |
| 38 | 7°30' 0,000" S | 70°15' 0,000" W |
| 39 | 3°28' 16,228" S | 68°56' 6,301" W |
| 40 | 3°43' 59,880" S | 62°25' 0,120" W |
| 41 | 10°46' 23,000" S | 53°5' 23,000" W |
| 42 | 0°15' 0,000" S | 65°55' 12,000" W |
| 43 | 10°26' 19,888" S | 62°28' 21,201" W |
| 44 | 17°1' 0,001" S | 39°33' 0,000" W |
| 45 | 10°56' 30,660" S | 68°51' 8,802" W |
| 46 | 4°22' 5,902" S | 70°2' 16,001" W |
| 47 | 4°20' 0,000" S | 60°1' 0,001" W |
| 48 | 8°24' 21,600" S | 71°19' 48,000" W |
| 49 | 12°0' 2,999" S | 38°18' 0,000" W |
| 50 | 14°10' 1,920" S | 39°43' 19,920" W |
| 51 | 9°37' 12,000" S | 55°13' 12,000" W |
| 52 | 0°57' 50,501" S | 46°52' 42,499" W |
| 53 | 12°51' 49,151" S | 38°23' 47,490" W |
| 54 | 10°48' 20,002" S | 65°0' 29,002" W |
| 55 | 9°10' 8,000" S | 68°57' 4,000" W |
| 56 | 3°2' 34,145" S | 59°58' 8,789" W |
| 57 | 13°23' 39,984" S | 60°25' 21,000" W |
| 58 | 10°46' 23,016" S | 53°5' 22,992" W |
| 59 | 1°41' 8,030" S | 50°28' 51,297" W |
| 60 | 3°28' 16,226" S | 68°56' 6,302" W |
| 61 | 7°30' 34,522" S | 63°1' 19,178" W |
| 62 | 10°26' 19,889" S | 62°28' 21,202" W |
| 63 | 12°50' 25,804" S | 69°24' 22,327" W |
| 64 | 12°44' 5,647" S | 72°33' 27,961" W |
| 65 | 12°30' 38,761" S | 68°58' 44,879" W |
| 66 | 1°0' 19,253" S | 77°48' 52,268" W |
| 67 | 1°0' 7,085" S | 77°48' 52,646" W |
| 68 | 0°59' 43,696" S | 77°48' 57,964" W |
| 69 | 0°59' 43,033" S | 77°48' 57,262" W |
| 70 | 0°59' 38,839" S | 77°49' 1,560" W |
| 71 | 0°59' 35,869" S | 77°48' 55,876" W |
| 72 | 0°59' 19,147" S | 77°48' 44,204" W |
| 73 | 0°59' 14,237" S | 77°49' 17,177" W |
| 74 | 0°59' 11,857" S | 77°49' 11,989" W |
| 75 | 0°59' 11,472" S | 77°49' 12,079" W |
| 76 | 0°59' 4,589" S | 77°49' 3,184" W |
| 77 | 0°58' 47,604" S | 77°50' 6,720" W |
| 78 | 0°58' 44,659" S | 77°48' 55,444" W |
| 79 | 0°5' 8,772" S | 78°27' 56,059" W |
| 80 | 2°32' 19,903" N | 72°38' 22,978" W |
| 81 | 6°14' 6,947" N | 75°33' 56,905" W |
| 82 | 6°16' 10,247" N | 75°33' 48,067" W |
| 83 | 6°20' 34,559" N | 75°34' 44,328" W |
| 84 | 7°3' 59,123" N | 73°5' 15,785" W |
| 85 | 7°4' 6,193" N | 73°5' 23,658" W |
| 86 | 7°4' 52,914" N | 73°6' 45,176" W |
| 87 | 7°5' 48,764" N | 73°5' 54,528" W |
| 88 | 7°5' 51,666" N | 73°5' 53,588" W |
| 89 | 7°5' 54,269" N | 73°5' 53,509" W |
| 90 | 7°5' 54,874" N | 73°5' 53,203" W |
| 91 | 7°5' 55,993" N | 73°5' 53,084" W |
| 92 | 7°5' 56,130" N | 73°5' 53,020" W |
| 93 | 7°7' 7,507" N | 73°6' 17,705" W |
| 94 | 10°21' 15,210" N | 75°25' 44,756" W |
| 95 | 10°21' 18,166" N | 75°25' 48,763" W |
| 96 | 10°21' 19,490" N | 75°25' 47,248" W |
| 97 | 12°36' 18,886" S | 69°7' 26,371" W |
| 98 | 6°28' 56,921" S | 76°22' 21,680" W |
| 99 | 3°6' 49,140" S | 60°0' 52,326" W |
| 100 | 1°37' 23,567" S | 79°29' 34,800" W |
| 101 | 1°26' 47,170" S | 48°25' 40,462" W |
| 102 | 0°6' 29,171" S | 80°7' 3,839" W |
| 103 | 4°9' 4,975" N | 73°38' 15,684" W |
| 104 | 13°31' 4,217" S | 72°0' 15,822" W |
| 105 | 0°7' 22,508" S | 80°7' 17,591" W |
| 106 | 0°7' 17,868" S | 80°7' 16,266" W |
| 107 | 0°3' 16,920" S | 78°46' 38,964" W |
| 108 | 0°3' 5,026" S | 78°46' 26,458" W |
| 109 | 3°29' 17,999" N | 73°44' 42,000" W |
| 110 | 5°37' 47,672" N | 74°51' 3,953" W |
| 111 | 5°54' 1,328" N | 74°51' 21,557" W |
| 112 | 5°57' 48,341" N | 75°6' 7,697" W |
| 113 | 10°21' 18,000" N | 75°25' 45,995" W |
| 114 | 6°30' 53,035" S | 76°21' 50,375" W |
| 115 | 5°21' 4,007" N | 75°37' 7,511" W |
| 116 | 5°46' 4,102" N | 74°58' 16,399" W |
| 117 | 6°50' 47,040" N | 73°22' 30,360" W |
| 118 | 10°19' 8,587" N | 75°24' 42,379" W |
| 119 | 3°9' 3,398" N | 75°27' 47,902" W |
| 120 | 14°43' 16,000" S | 39°21' 58,000" W |
| 121 | 14°43' 16,000" S | 39°22' 4,001" W |
| 122 | 14°43' 14,002" S | 39°21' 58,000" W |
| 123 | 14°43' 13,001" S | 39°21' 56,999" W |
| 124 | 14°43' 13,001" S | 39°21' 58,000" W |
| 125 | 14°43' 12,000" S | 39°21' 56,002" W |
| 126 | 14°43' 10,999" S | 39°21' 55,001" W |
| 127 | 14°43' 7,000" S | 39°21' 51,001" W |
| 128 | 14°43' 0,998" S | 39°22' 13,001" W |
| 129 | 14°43' 0,998" S | 39°22' 4,001" W |
| 130 | 14°42' 56,999" S | 39°22' 5,999" W |
| 131 | 14°11' 21,152" S | 68°20' 2,141" W |
| 132 | 10°21' 15,001" S | 75°17' 6,000" W |
| 133 | 1°48' 42,872" S | 79°20' 10,507" W |
| 134 | 0°1' 4,865" S | 76°10' 26,731" W |
| 135 | 6°4' 12,832" N | 77°23' 34,242" W |
| 136 | 6°5' 16,127" N | 77°24' 53,654" W |
| 137 | 6°6' 14,922" N | 77°25' 40,019" W |
| 138 | 12°35' 14,075" S | 69°10' 34,226" W |
| 139 | 2°35' 44,830" S | 77°20' 30,232" W |
| 140 | 0°53' 27,787" S | 75°27' 14,742" W |
| 141 | 7°54' 34,319" N | 76°36' 3,150" W |
| 142 | 8°35' 55,136" N | 73°13' 24,488" W |
| 143 | 3°47' 17,520" N | 76°23' 30,458" W |
| 144 | 14°46' 52,799" S | 39°16' 21,500" W |
| 145 | 14°48' 0,000" S | 39°10' 0,001" W |
| 146 | 12°32' 38,760" S | 70°6' 18,360" W |
| 147 | 8°16' 27,264" N | 76°13' 32,520" W |
| 148 | 10°12' 0,000" S | 75°19' 59,988" W |
| 149 | 3°43' 9,998" S | 70°18' 25,999" W |
| 150 | 1°51' 15,001" N | 75°42' 24,001" W |
| 151 | 1°27' 59,976" S | 78°3' 0,000" W |
| 152 | 1°27' 0,000" S | 79°16' 59,988" W |
| 153 | 1°35' 37,702" N | 75°38' 54,949" W |
| 154 | 2°25' 48,000" N | 74°35' 24,000" W |
| 155 | 3°25' 51,290" N | 76°33' 35,996" W |
| 156 | 12°3' 0,000" S | 38°18' 0,000" W |
| 157 | 1°39' 0,000" N | 75°36' 24,001" W |
| 158 | 13°33' 59,976" S | 68°39' 59,976" W |
| 159 | 11°31' 0,001" S | 69°6' 0,000" W |
| 160 | 11°19' 59,880" S | 67°40' 0,120" W |
| 161 | 5°19' 15,996" S | 78°41' 4,992" W |
| 162 | 2°15' 43,200" S | 77°12' 28,800" W |
| 163 | 1°55' 8,400" S | 77°49' 33,600" W |
| 164 | 0°37' 59,988" S | 76°9' 59,976" W |
| 165 | 14°13' 12,000" S | 69°10' 12,000" W |
| 166 | 10°4' 12,000" S | 71°6' 0,000" W |
| 167 | 9°53' 0,000" S | 69°31' 0,001" W |
| 168 | 9°53' 0,000" S | 69°31' 12,000" W |
| 169 | 14°45' 0,000" S | 66°1' 48,000" W |
| 170 | 14°45' 0,000" S | 66°20' 0,000" W |
| 171 | 5°18' 59,976" S | 78°46' 59,988" W |
| 172 | 14°21' 59,976" S | 67°33' 0,000" W |
| 173 | 8°24' 23,000" S | 71°19' 54,001" W |
| 174 | 8°24' 21,600" S | 71°19' 55,200" W |
| 175 | 6°15' 0,000" N | 74°34' 12,000" W |
| 176 | 8°16' 44,000" S | 71°5' 16,001" W |
| 177 | 10°7' 0,001" S | 69°13' 0,001" W |
| 178 | 4°51' 0,000" S | 73°50' 0,000" W |
| 179 | 0°49' 0,001" S | 76°22' 0,001" W |
| 180 | 3°42' 0,000" S | 70°15' 0,000" W |
| 181 | 2°24' 0,000" S | 79°36' 0,000" W |
| 182 | 1°29' 43,944" N | 78°39' 40,392" W |
| 183 | 16°7' 9,998" S | 66°0' 6,001" W |
| 184 | 3°47' 0,000" S | 70°17' 0,000" W |
| 185 | 3°46' 59,988" S | 70°15' 0,000" W |
| 186 | 3°23' 58,524" S | 70°8' 30,912" W |
| 187 | 1°25' 0,001" S | 77°20' 0,000" W |
| 188 | 0°58' 12,000" S | 77°27' 0,000" W |
| 189 | 0°58' 0,001" S | 77°27' 0,000" W |
| 190 | 10°28' 59,988" N | 66°54' 0,000" W |
| 191 | 13°34' 59,988" S | 68°45' 59,976" W |
| 192 | 13°34' 59,880" S | 68°46' 0,120" W |
| 193 | 3°30' 0,000" S | 72°49' 59,988" W |
| 194 | 5°1' 48,475" N | 75°26' 2,296" W |
| 195 | 15°27' 59,976" S | 67°27' 59,976" W |
| 196 | 14°30' 0,000" S | 67°30' 0,000" W |
| 197 | 12°50' 0,000" S | 69°17' 0,000" W |
| 198 | 12°49' 59,988" S | 69°16' 59,988" W |
| 199 | 12°49' 48,000" S | 69°16' 48,000" W |
| 200 | 12°4' 59,988" S | 69°3' 0,000" W |
| 201 | 11°12' 0,000" S | 69°20' 0,000" W |
| 202 | 9°40' 12,000" S | 75°1' 48,000" W |
| 203 | 9°40' 0,001" S | 75°2' 0,000" W |
| 204 | 3°30' 0,000" S | 78°10' 12,000" W |
| 205 | 3°30' 0,000" S | 78°10' 0,001" W |
| 206 | 0°9' 59,976" N | 79°34' 59,988" W |
| 207 | 17°45' 0,000" S | 64°45' 0,000" W |
| 208 | 11°10' 0,001" S | 69°7' 0,001" W |
| 209 | 11°9' 59,976" S | 67°6' 59,976" W |
| 210 | 1°24' 52,600" N | 78°21' 32,198" W |
| 211 | 1°45' 0,000" N | 67°2' 0,000" W |
| 212 | 1°55' 0,001" N | 67°2' 0,000" W |
| 213 | 2°25' 0,001" N | 64°14' 0,000" W |
| 214 | 2°36' 0,000" N | 66°12' 0,000" W |
| 215 | 3°45' 0,000" N | 64°59' 0,000" W |
| 216 | 4°47' 0,000" N | 65°24' 0,000" W |
| 217 | 5°3' 35,237" N | 75°34' 30,072" W |
| 218 | 14°30' 0,000" S | 66°37' 0,001" W |
| 219 | 14°28' 0,001" S | 67°34' 0,008" W |
| 220 | 13°45' 0,000" S | 68°9' 0,000" W |
| 221 | 11°10' 59,988" S | 68°40' 59,988" W |
| 222 | 4°49' 59,988" S | 73°45' 0,000" W |
| 223 | 6°55' 0,001" N | 68°42' 0,000" W |
| 224 | 7°4' 7,050" N | 73°5' 17,700" W |
| 225 | 7°4' 11,064" N | 73°5' 22,488" W |
| 226 | 7°39' 4,000" N | 67°1' 40,004" W |
| 227 | 2°45' 0,000" S | 76°30' 0,000" W |
| 228 | 1°28' 29,424" N | 78°37' 58,692" W |
| 229 | 3°3' 0,000" N | 65°27' 0,004" W |
| 230 | 3°10' 0,001" N | 65°33' 0,000" W |
| 231 | 3°24' 9,666" N | 65°37' 42,092" W |
| 232 | 3°30' 0,000" N | 65°12' 0,004" W |
| 233 | 6°34' 0,001" N | 66°59' 0,000" W |
| 234 | 3°39' 59,976" S | 73°30' 0,000" W |
| 235 | 5°41' 35,520" N | 76°39' 5,184" W |
| 236 | 11°51' 59,976" S | 71°21' 59,976" W |
| 237 | 10°50' 2,040" S | 52°52' 23,160" W |
| 238 | 4°2' 0,000" S | 59°6' 0,000" W |
| 239 | 4°1' 59,988" S | 59°6' 0,000" W |
| 240 | 4°1' 59,880" S | 59°6' 0,000" W |
| 241 | 0°31' 0,001" S | 76°23' 0,000" W |
| 242 | 6°2' 54,316" N | 75°0' 5,922" W |
| 243 | 6°4' 58,728" N | 74°50' 49,884" W |
| 244 | 11°18' 8,708" N | 74°10' 13,080" W |
| 245 | 12°4' 0,001" S | 70°33' 0,000" W |
| 246 | 2°53' 0,000" S | 64°52' 0,001" W |
| 247 | 0°31' 0,120" S | 76°22' 59,880" W |
| 248 | 0°21' 0,000" S | 79°21' 59,976" W |
| 249 | 0°21' 0,000" S | 79°21' 57,600" W |
| 250 | 0°48' 0,000" N | 78°54' 0,000" W |
| 251 | 6°55' 36,383" N | 75°34' 17,454" W |
| 252 | 5°36' 45,551" N | 75°31' 59,077" W |
| 253 | 7°34' 59,880" N | 72°7' 59,880" W |
| 254 | 7°34' 59,988" N | 72°7' 59,988" W |
| 255 | 7°45' 0,000" N | 71°28' 59,988" W |
| 256 | 9°41' 0,000" S | 67°39' 0,000" W |
| 257 | 0°16' 59,988" S | 75°52' 59,988" W |
| 258 | 0°16' 58,800" S | 75°52' 58,800" W |
| 259 | 0°15' 0,000" S | 76°21' 0,000" W |
| 260 | 0°6' 57,600" N | 76°54' 57,600" W |
| 261 | 0°6' 59,976" N | 76°54' 59,976" W |
| 262 | 0°7' 0,001" N | 76°55' 0,001" W |
| 263 | 11°11' 0,000" S | 69°2' 0,000" W |
| 264 | 7°28' 17,652" N | 73°20' 41,208" W |
| 265 | 8°39' 59,976" S | 75°0' 0,000" W |
| 266 | 3°44' 52,980" S | 73°14' 49,992" W |
| 267 | 4°58' 0,001" N | 74°26' 0,000" W |
| 268 | 14°0' 0,000" S | 65°30' 0,000" W |
| 269 | 13°51' 54,000" S | 66°47' 7,998" W |
| 270 | 12°0' 0,000" S | 70°15' 0,000" W |
| 271 | 3°19' 0,001" S | 71°51' 0,000" W |
| 272 | 0°53' 34,980" S | 79°29' 20,976" W |
| 273 | 4°27' 59,976" S | 78°9' 0,000" W |
| 274 | 4°59' 20,911" N | 75°39' 39,823" W |
| 275 | 4°47' 59,381" N | 75°50' 57,768" W |
| 276 | 5°0' 0,400" N | 75°35' 12,005" W |
| 277 | 9°4' 12,000" S | 68°37' 12,000" W |
| 278 | 9°4' 0,001" S | 68°37' 0,001" W |
| 279 | 7°15' 20,365" S | 64°47' 54,978" W |
| 280 | 2°22' 59,880" S | 77°30' 0,000" W |
| 281 | 7°4' 32,880" N | 65°54' 42,840" W |
| 282 | 2°48' 38,815" N | 73°48' 13,583" W |
| 283 | 3°44' 0,000" S | 62°25' 0,001" W |
| 284 | 4°39' 3,013" N | 74°27' 58,061" W |
| 285 | 4°24' 19,267" S | 69°55' 32,563" W |
| 286 | 6°13' 31,501" N | 77°24' 13,637" W |
| 287 | 1°41' 8,030" S | 50°28' 51,297" W |
| 288 | 11°3' 0,000" S | 75°19' 0,120" W |

**References**

1. SpeciesLink; 2019 [cited 2019 Oct 17]. Database: Theobroma cacao L. collection - Occurrence data [Internet]. Available from: <http://www.splink.org.br/index?lang=pt>.
2. Global Biodiversity Information Facility; 2019 [cited 2019 Oct 17]. Database: Theobroma cacao L. collection - Occurrence data [Internet]. Available from: <https://doi.org/10.15468/dl.l5kuzh>.
